# Sildenafil citrate increases myocardial cGMP content in rat heart, decreases its hypertrophic response to isoproterenol and decreases myocardial leak of creatine kinase and troponin T

**DOI:** 10.1186/1471-2210-5-10

**Published:** 2005-04-06

**Authors:** Madiha AH Hassan, Amal F Ketat

**Affiliations:** 1 Department of Pharmacology, Faculty of Medicine, University of Alexandria, Egypt; 2 Department of Medical Biochemistry, Faculty of Medicine, University of Alexandria, Egypt

## Abstract

**Background:**

Cardiac hypertrophy is a major risk factor for morbidity and mortality in a number of cardiovascular diseases. Consequently, the signaling pathways that inhibit cardiac hypertrophy are currently receiving much interest. Among them, nitric oxide (NO), signaling via cGMP and cGMP-dependent protein kinase I, has been recognized as a negative regulator of cardiac hypertrophy. The present study investigated the in-vivo effect of sildenafil as a phosphodiestrase-5A (PDE-5A) inhibitor on the hypertrophic response of rat heart to isoproterenol and the relation of this effect to the level of myocardial cGMP and integrity of the constitutive nitric oxide synthase (cNOS) activity.

**Results:**

The results showed that daily intraperitoneal administration of sildenafil per se for 10 days was without noticeable adverse effects on survival or myocardium. Conversely, daily subcutaneous administration of isoproterenol for 10 days caused significant myocardial hypertrophy, cell injury and decline in survival. When sildenafil was injected daily, one hour before isoproterenol, survival was significantly improved and the myocardium didn't show significant hypertrophy or cell injury. Interestingly, sildenafil was accompanied by significant rise in myocardial cGMP level, a parameter which was found in the present study to possess a significant negative correlation with cardiac hypertrophy and leak of cardiac troponin T into serum. At the same time, cGMP was found to possess a positive correlation with myocardial creatine kinase activity that reflects the efficiency of the energy utilization processes in the myocardium. However, in rats given N^ω^-nitro-L-arginine (L-NNA) as a competitive inhibitor of cNOS, sildenafil failed to show any favorable effect on survival or the myocardial injury parameters used to assess isoproterenol-induced injury.

**Conclusion:**

The present study suggests that increased cardiac cGMP level by sildenafil have a cardioprotective effect probably through acting as a post-receptor negative regulator of cardiac sympathetic responsiveness. Integrity of NOS function was an essential prerequisite for sildenafil's mediated cardioprotection encountered in the present study.

## Background

Sildenafil is a selective inhibitor of phosphodiesterase-5A (PDE-5A), the enzyme that hydrolyzes cGMP. Sildenafil is orally effective in the treatment of erectile dysfunction. Its pharmacological action is due to prolonging the signaling actions of nitric oxide (NO) in penile smooth muscle [1]. Interestingly, a recent publication reported a pronounced infarct size-reducing effect of sildenafil in an in-vivo rabbit model of coronary occlusion [2]. Reduced infarct size by sildenafil has also been reported in mice and rat heart subjected to global ischemic/reperfusion (I/R) injury [3,4]. In those studies, opening of mitochondrial ATP-sensitive K channels, induction of NO synthase (NOS) isoforms and increased cGMP level by sildenafil were suggested to mediate a preconditioning-like cardioprotective effect [2-4].

As regards myocardial hypertrophy, a great number of in-vivo and in-vitro studies tested the role of many signaling pathways involved in its induction [5]. At the same time, the role of the negative regulators such as NO, atrial natriuretic peptide and cGMP have so far received much less attention until recently recognized to be of particular significance [6,7]. Alternatively, drugs that modulate the NO-cGMP signaling pathway like sildenafil and probably its congeners may be useful especially that sildenafil proved to be cardioprotective in models of cardiac I/R injury.

To address this issue, the present study examined the cardioprotective effect of sildenafil in a rat model of ventricular hypertrophy and myocardial cell injury induced by the β-adrenergic agonist isoproterenol. The model is characterized by technical simplicity, excellent reproducibility and an acceptable level of mortality [8]. Previous studies in rats revealed that isoproterenol induced a dose-dependent increase in ventricular end diastolic pressure and global wall stress that were associated with compensatory hypertrophy and repair fibrosis [5,9]. However, in the present study, the following parameters were assessed: rate of survival among experimental groups, heart coefficient, myocardial cGMP level, myocardial creatine kinase activity and serum level of cardiac troponin T (cTnT). In addition, the role of constitutive NOS was examined by adding the NOS inhibitor N^ω^-nitro-L-arginine (L-NNA) to drinking water of a group of sildenafil and isoproternol treated rats.

## Results

### Cumulative survival curves (Fig. 1)

**Figure 1:**
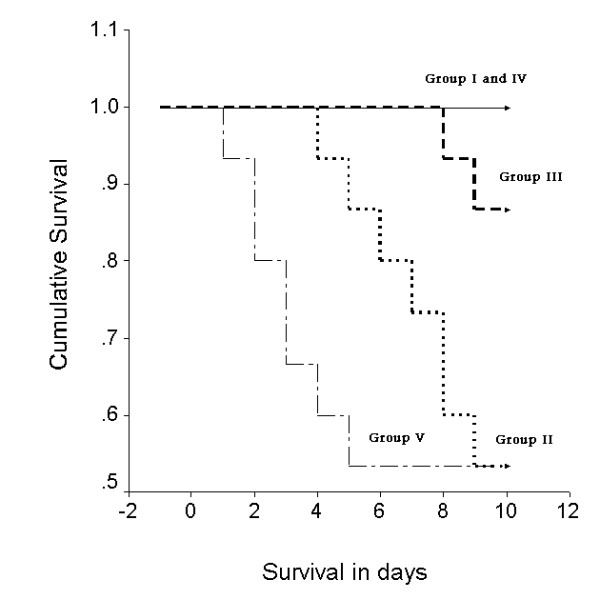
Kaplan-Meier analysis of 10 days survival among 70 rats classified into vehicle (group I, n= 10; solid line), isoproterenol (group II, n = 15; thick dotted intermediate curve), isoproterenol/ sildenafil (group III, n = 15; thick dashed curve), sildenafil/vehicle(group IV, n = 15; solid line) and sildenafil/isoproternol/ Nω-nitro-L-arginine (group V, n = 15; thin dash-dot line to the left). Significant improvement in survival rate was found by log-rank test in group III compared to groups II and V (P < 0.05).

In group I (vehicle control) and group IV (sildenafil/vehicle), survival was 100 % till termination of study (10 days). In group II (isoproterenol) and group V (sildenafil/isoproternol/L-NNA), survival was 53.33% (8/15) in each group. In group III (sildenafil/isoproternol) survival was 86.67% (13/15). By log-rank test, significant improvement in survival rate was found in group III compared to groups II and V (P < 0.05).

### Heart coefficient, myocardial creatine kinase (CK) activity, myocardial cGMP level and serum cardiac troponin T (cTnT)

Table I shows significant increase in heart coefficient in group II (isoproterenol) and group V (sildenafil/isoproterenol/L-NNA) compared to group I (vehicle control), group III (sildenafil/isoproternol) and group IV (sildenafil/vehicle) denoting occurrence of cardiac hypertrophy in the former groups. Also, significant myocardial cell injury in group II and group V was indicated by the decline of myocardial CK activity and leak of cTnT into serum in these groups compared to group I. At the same time, myocardial CK activity and serum level of cTnT in group III and group IV were normal and comparable to group I (homogenous subset). Elevated myocardial cGMP was found in two sildenafil groups (III and IV) compared to other groups (I, II and V).

**Table 1:** The heart coefficient (mg/g body wt.), myocardial cGMP level (pmoles/g wet tissue), CK activity (U/mg tissue protein) and serum level of cTnT (ng/ml) in rats after 10 days of treatment with: vehicle (group I), isoproterenol (group II), sildenafil/ isoproterenol (group III), sildenafil (group IV) and sildenafil/isoproternol/ N^ω^-nitro-L-arginine (group V).

	**Heart coefficient**	**Myocardial cGMP**	**Myocardial CK**	**Serum cTnT**
**Group I n = 10**	3.33 ± 0.18*	4.76 ± 1.03**	6.03 ± 0.35**	0.38 ± 0.13*
**Group II n = 8**	5.27 ± 0.17**	4.12 ± 1.36**	3.10 ± 0.35*	6.50 ± 1.29***
**Group III n = 13**	3.75 ± 0.48*	10.51 ± 3.05***	5.62 ± 0.44**	3.71 ± 1.16**
**Group IV n = 15**	3.49 ± 0.67*	10.14 ± 3.24***	5.80 ± 1.02**	0.75 ± 0.28*
**Group V n = 8**	5.31 ± 0.47**	1.80 ± 0.54*	3.47 ± 0.37*	6.34 ± 1.14***

NB: Isoproterenol was given at the dose of 5 mg/kg daily s.c. and sildenafil was given at the dose of 0.7 mg/kg daily i.p. and N^ω^-nitro-L-arginine was added to drinking water at the concentration of 10 mg/L.

Values are mean ± SEM. n: number of rats survived for 10 days

One-way ANOVA test followed by Student-Newman-Keuls post-hoc test. Values forming homogeneous subsets are as follow: *homogeneous subset 1, ** homogeneous subset 2 and *** homogeneous subset 3. Significant difference existed between different subsets (P < 0.05)

### Correlation of myocardial cGMP level to other parameters

**Figure 2:**
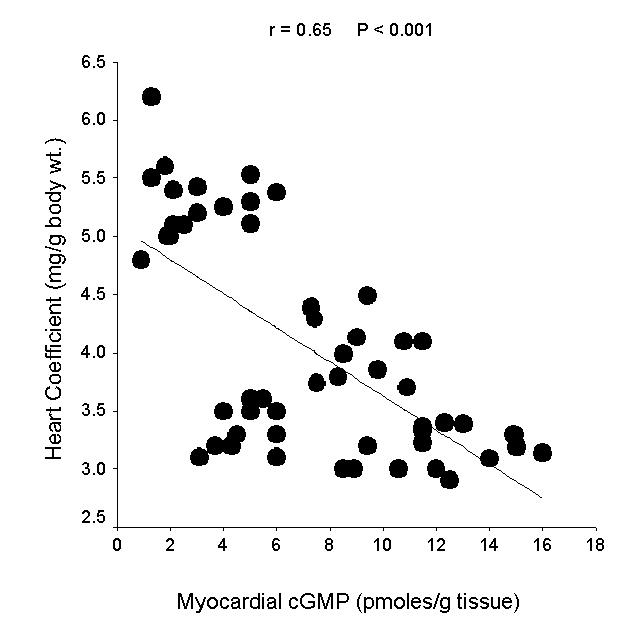
Linear regression curve for myocardial cGMP level and heart coefficient.

**Figure 3:**
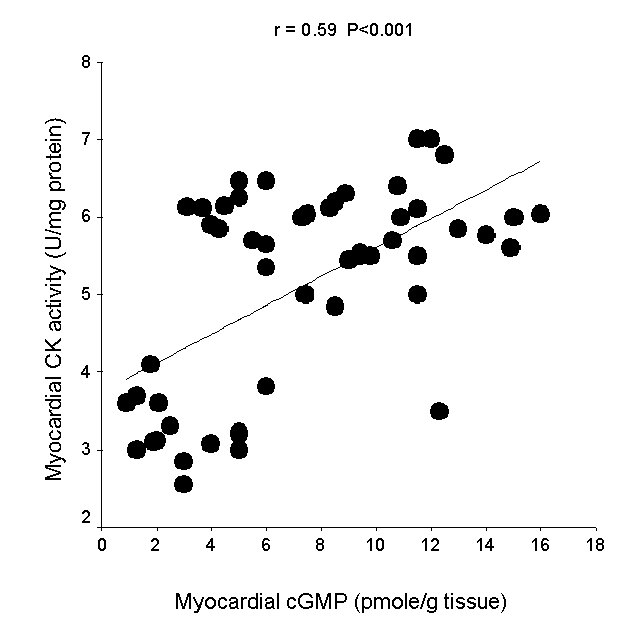
Linear regression curve for myocardial cGMP and myocardial creatine kinase (CK) activity.

**Figure 4:**
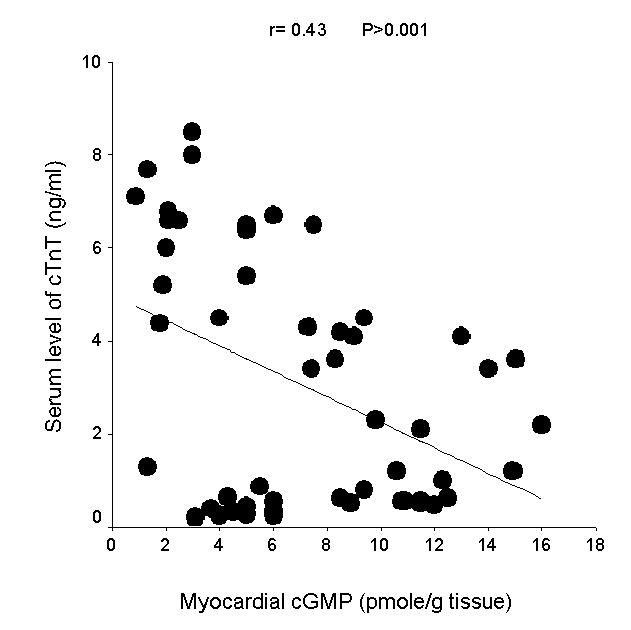
Linear regression curve for myocardial cGMP and serum level of cardiac troponin T (cTnT).

The linear regression curves shown in the figures 2, 3, 4, demonstrate significant correlations between myocardial cGMP level and heart coefficient (r = - 0.65, P < 0.001), myocardial CK activity (r = 0.59, P < 0.001) and serum cTnT level (r = - 0.43, P < 0.01). Individual data from all studied rats were included in the correlation analysis.

## Discussion

Dealing with cardiac hypertrophy is currently a major goal of cardiovascular research and clinical trials[5]. The cardiomyocytes undergo hypertrophy in some pathological conditions that impose overwork on the heart e.g. hypertension, heart valve diseases, myocardial infarction, and cardiomyopathy [16]. Such cardiac hypertrophy is initially compensatory for an increased work load, however, prolongation of this process eventually leads to congestive heart failure, arrhythmia, and sudden death [16]. Todate, drugs targeting the renin-angiotensin system are the chief class of cardiovascular agents of special clinical utility in settings predisposing to cardiac hypertrophy based on the important role of angiotensin II in growth of cardiomyocytes [17].

In an attempt to examine other signaling pathways and other drugs, the present study probed the NO-cGMP pathway and the effect of its modulation by the selective PDE-5A inhibitor sildenafil in cardiac hypertrophy. The β-adrenergic agonist isoproterenol was subcutaneously injected in rats for 10 days to induce cardiac hypertrophy. Obtained results showed that sildenafil administration one hour before daily s.c. injection of isoproterenol was associated with significant improvement in survival and significant inhibition of cardiac hypertrophy. Also, the myocardium of the sildenafil-isoproterenol treated rats showed higher activity of myocardial CK activity and less cTnT leaked into the blood compared to isoproterenol-treated rats. These findings suggest that sildenafil conferred a significant anti-hypertrophic and cytoprotective effect on cardiomyocytes. Conversely, the decline in myocardial CK activity and increased leak of cTnT into serum observed in the isoproterenol treated rats and in rats subjected to NOS inhibition denote significant impairment in tissue energy metabolism and loss of cell integrity, respectively. In the myocardium, ATP is synthesized mainly in the mitochondria through oxidative phosphorylation and transported to the contractile apparatus, where it is consumed by myosin ATPase to generate force. The creatine kinase system plays an important role in myocardial energy metabolism by maintaining ADP levels high at the mitochondria, where ATP is generated, and low at sites of ATP utilization [18]. This is postulated to contribute to the maintenance of a high free energy of ATP hydrolysis, thereby enhancing the efficiency of the energy utilization processes [18]. In addition, a CK shuttle has been proposed, in which high-energy phosphate transport within the cell is facilitated by the higher diffusibility of creatine and phosphocreatine relative to ADP [18].

As regards cardiac troponins T, I and C, they regulate muscle contraction by modulating calcium-dependent interaction of actin and myosin. These intracellular structural proteins are released into circulation following loss of cell integrity [19].

An interesting observation in the present study was that, in the presence of the cNOS inhibitor L-NNA, sildenafil was deprived from its antihypertrophic and cytoprotective effects in isoproterenol-treated rats. Also, the significant correlations found between tissue cGMP level on one hand and heart coefficient, myocardial CK activity and serum level of cTnT on the other hand, suggest that the integrity of the NO-cGMP signaling pathway plays a pivotal role in the cardioprotective effect of sildenafil.

The second messenger cGMP that was identified almost 40 years ago, is generated from GTP either by soluble guanyl cyclase or particulate guanyl cyclase [20]. The former is activated by nitric oxide (NO) or carbon monoxide, where as the latter binds a family of natriuretic peptides consisting of atrial, brain, and C-type natriuretic peptides [20]. On the other hand, various phosphodiesterases regulate cGMP catabolism, including PDE-5A, PDE-6, PDE-9A, PDE-10A, and PDE-11A according to their tissue specificity [21-23]. Among these, PDE-5A is the most widely studied, and its inhibition is a primary mechanism for efficacy of sildenafil in erectile dysfunction [1]. Previous studies have shown that inhibition of PDE-5A in the myocardium enhanced coronary blood flow during exercise-induced ischemia, blunted cardiac stimulation by dobutamine and reduced contractility of adrenergically stimulated papillary muscle [22-24]. In accordance with this, inhibition of PDE-5A in rat myocardium probably underlies the cardioprotective effect of sildenafil against isoproterenol-induced cardiac hypertrophy observed in the present study. At cellular level, the common denominator in all these studies is probably the increased cellular level of cGMP.

In non stimulated hearts, cGMP has been suggested to augment contractile function at low concentrations, likely via cross-talk with cAMP-dependent signaling, inhibiting PDE-3 and degradation of cAMP [25]. At higher concentrations, cGMP has a negative inotropic effect by antagonizing cAMP via protein kinase G (in mammals) or PDE-2 stimulation (in amphibians) [25]. With β-adrenergic activation, both cAMP and cGMP synthesis increase, with the net effect of cGMP being negative on the inotropic response (a brake) [26]. Conversely, reducing cGMP level e.g. by NOS inhibition, enhances β-adrenergic responsiveness [27]. In addition to the negative inotropic action of cGMP, it reduces oxygen consumption and offsets the development of cardiac hypertrophy [28,29].

Consequently, PDE-5A inhibition by sildenafil and cellular accumulation of cGMP would be the braking force against isoproterenol-induced cardiac hypertrophy found in the present study. Consistent with this assumption, over-expression of the catalytic fragment of the constitutively active guanylate cyclase domain of the atrial natriuretic peptide receptor in mouse heart increased the intracellular concentration of cGMP within cardiomyocytes and attenuated the effects of isoproterenol on cardiac wall thickness and prevented fetal gene expression program normally associated with cardiac hypertrophy [30]. Conversely, disruption of cardiac guanylate cyclase-A (GC-A) gene resulted in mice that displayed elevation of blood pressure, cardiac fibrosis and hypertrophy [31].

## Conclusion

The present study suggests that sildenafil possesses a cardioprotective and antihypertrophic effect against isoproterenol-induced myocardial injury. Inhibition of cGMP degradation by sildenafil with a consequent accumulation of this signaling molecule may act as a negative regulator against cardiac hypertrophy in-vivo. Understanding the downstream molecular mechanism(s) of such effect of sildenafil, may expand the utility of this drug beyond the current use for treatment of erectile dysfunction in men. The study also revealed that integrity of function of cNOS is an essential prerequisite for the cardioprotective effect of sildenafil in the adrenergically stimulated heart.

## Methods

### Experimental animals

The present study was conducted on 70 male albino rats of 5 months age, weighing 200–230 g/rat, from those bred in the animal house of the Pharmacology Department, Faculty of Medicine, Alexandria University, Egypt. Rats were kept in galvanized iron cages in groups of 2–3 rats/cage, at room temperature of 22–25°C and allowed free access to standard chow diet and tap water. The study was carried out in accordance with the local guidelines for animal experimentation.

### Drugs, chemicals and kits

Isoproterenol and chemicals were purchased from Sigma-Aldrich, Inc. (USA). Sildenafil tablets of Pfizer Inc. (Viagra 100 mg/tablet) were crushed and dissolved in saline for intraperitoneal injection. Direct cGMP enzyme immunoassay kit (CG-200, Sigma-Aldrich, Inc. USA) was used to measure myocardial cGMP level. Cardiac troponin T enzyme immunoassay kit (ES300, Boehringer Mannheim Immunodiagnostics, Germany) was used to measure serum cTnT.

N^ω^-nitro-L-arginine (L-NNA) was purchased from Cayman chemical, USA.

### Experimental groups and treatment

Rats were randomly assigned into 5 groups:

**Group I **(*n = 10*)**: **A control group that received vehicle (saline) instead of drug injection.

**Group II ***(n = 15)***: **Isoproterenol was injected s.c. at a dose of 5 mg/kg/day [10].

**Group III ***(n = 15)*: Sildenafil was injected i.p. at a dose of 0.7 mg/kg/day, one hour prior to isoproterenol. The drug was given in an experimental dose approximating, on a mg/kg basis, the clinical dose of 50 mg administered to a 70-kg patient as described by Okcaili et al [2].

**Group IV ***(n = 15)*: Sildenafil was injected i.p. at mentioned dose, one hour prior to s.c. injection of saline instead of isoproterenol. This group served as a control for Group III.

**Group V ***(n = 15)*: Drugs given to this group were essentially similar to group III, however, the NOS inhibitor N^ω^-nitro-L-arginine (L-NNA) was added to drinking water of this group at the concentration of 10 mg/L. This concentration was selected on the previously reported insignificant effect on blood pressure in rats [11].

### Determination of survival, serum level of cTnT and heart coefficient

The study continued for 10 days [10]. The number of survivors in each group was recorded daily and on day 11^th^, 3 ml-trunkal blood sample/rat was collected under light ether anesthesia from the inferior vena cava and centrifuged for 15 min at 3000 r.p.m. Sera were kept frozen at -20°C until assayed for cardiac troponin T according to general principles of ELISA technique [12] and the manufacturer's instructions (Boehringer- Mannheim Immunodiagnostics, Germany), with a lower limit of detection of 0.01 ng/ml. The means ± SEM were determined for triplicate samples.

From each rat, the heart was excised immediately after rat sacrifice and weighed to calculate the ratio of heart weight to body weight (heart coefficient) [10].

### Determination of myocardial creatine kinase (CK) activity

Tissue extract of apical myocardium was prepared by homogenization in cold 20 mM Tris/HCl buffer at the ratio of 1:3 (w/v), pH 7.5, containing 1 mM EDTA and 1 mM β-mercaptoethanol. Cellular debris were removed by centrifugation at 4°C for 60 minutes at 15.000 r.p.m. and the supernatant was used for the assay of CK activity spectrophotometrically at 340 nm, as recommended by the International Federation of Clinical Chemistry [13]. Creatine kinase is primarily concerned with ATP regeneration through catalyzing the following reaction:

Adenosine diphosphate (ADP) + phosphocreatine  ATP + Creatine.

The present assay of CK activity was based on the formation of ATP linked to the production of NADHP via hexokinase and glucose-6-phosphate dehydrogenase. The reaction mixture contained 100 mM imidazole acetate, 2 mM EDTA, 10 mM magnesium acetate, 2 mM ADP, 5 mM AMP, 20 mM D-glucose, 2 mM NADP, 30 mM phosphocreatine, hexokinase (3 U/ml), and glucose-6-phosphate dehydrogenase (2 U/ml), pH 6.7. One unit CK activity is that converts one micromole of creatine phosphate to creatine per minute at the given pH.

The protein concentration in supernatant tissue extract was determined by Lowry method [14].

### Determination of myocardial cGMP level

Frozen myocardial tissue samples in liquid nitrogen were ground to a fine powder in a stainless steel mortar. After the liquid nitrogen was evaporated, the frozen tissue was weighed and homogenize in 10 volumes of 0.1 M HCl to stop the action of phosphodiesterases. Centrifugation was done at 30.000 r.p.m. at room temperature and the supernatant was then collected for quantitative immunoassay of cGMP level according to general principle of ELISA technique [12] and the manufacturer's instructions (CG-200, Sigma-Aldrich, Inc. USA). The assay used a polyclonal antibody to cGMP to bind, in a competitive manner, cGMP in samples and standards or cGMP covalently attached to alkaline phosphatase. After incubation with the p-nitrophenyl phosphate substrate, a microplate autoreader (Bio-Tek Instruments EL311) was used to measure the intensity of the bound yellow colour at 405 nm. The measured optical density was used to calculate the concentration of cGMP in samples by interpolation from Logit-Log paper plot of the percent bound (B/Bo) versus concentration of cGMP for the standards.

### Statistical analysis

Data were analyzed by SPSS/PC, version 9 Chicago/L software. The survival curves were generated using the method of Kaplan and Meier, and the log-rank test was used to detect significant difference between survival curves. Values of other parameters were expressed as mean ± SEM (standard error of mean). Other parameters were compared by one-way ANOVA, with post-hoc comparisons by Student Newman-Keuls test. Correlation studies were carried out by linear regression with curve estimation. For all of the determinations, P < 0.05 was used to indicate statistical significance [15].

## Abbreviations

ADP, adenosine diphosphate; ATP, adenosine triphosphate; cAMP, cyclic adenosine monophosphate; cGMP: cyclic guanosine monophosphate; CK, creatine kinase; cTnT: cardiac troponin T; GC, guanyl cyclase; s.c., subcuteanous; i.p., intraperitoneal; NO, nitric oxide; cNOS, constitutive nitric oxide synthase; PDE, phosphodiesterase; w/v, weight/volume.

## Authors' contributions

MAHH designed the study, supervised drug administration and collection of samples, estimated the heart coefficient and participated in the biochemical procedures for cGMP, CK and cTnT measurement. MAHH also performed the statistical analysis and prepared the manuscript. AFK participated in the biochemical procedures and in the preparation of the manuscript.
